# Место двусторонней ревизии шеи при хирургическом лечении первичного гиперпаратиреоза

**DOI:** 10.14341/probl13096

**Published:** 2022-12-20

**Authors:** Д. М. Бузанаков, И. В. Слепцов, А. А. Семенов, Р. А. Черников, К. Ю. Новокшонов, Ю. В. Карелина, Н. И. Тимофеева, Л. Г. Яневская, Т. А. Джуматов

**Affiliations:** Клиника высоких медицинских технологий им. Н. И. Пирогова Санкт-Петербургского Государственного Университета; Клиника высоких медицинских технологий им. Н. И. Пирогова Санкт-Петербургского Государственного Университета; Клиника высоких медицинских технологий им. Н. И. Пирогова Санкт-Петербургского Государственного Университета; Клиника высоких медицинских технологий им. Н. И. Пирогова Санкт-Петербургского Государственного Университета; Клиника высоких медицинских технологий им. Н. И. Пирогова Санкт-Петербургского Государственного Университета; Клиника высоких медицинских технологий им. Н. И. Пирогова Санкт-Петербургского Государственного Университета; Клиника высоких медицинских технологий им. Н. И. Пирогова Санкт-Петербургского Государственного Университета; Национальный медицинский исследовательский центр им. В.А. Алмазова; Санкт-Петербургский государственный университет

**Keywords:** первичный гиперпаратиреоз, двусторонняя ревизия шеи, селективная паратиреоидэктомия, предоперационная визуализация

## Abstract

ОБОСНОВАНИЕ. Предоперационная визуализация околощитовидных желез имеет важное значение для эффективного лечения первичного гиперпаратиреоза (ПГПТ). Несмотря на то что большая часть пациентов с ПГПТ может быть вылечена путем выполнения селективной паратиреоидэктомии, планируемой на основании данных предоперационной визуализации, при таком подходе у значимого числа пациентов в послеоперационном периоде наблюдется персистенция гиперпаратиреоза, обусловленная наличием множественного поражения околощитовидных желез.ЦЕЛЬ. Целью настоящего исследования являлось изучение возможностей предоперационной визуализации около-щитовидных желез при планировании объема хирургического вмешательства при лечении больных с ПГПТ.МАТЕРИАЛЫ И МЕТОДЫ. Ретроспективное когортное исследование было проведено на выборке пациентов, первично прооперированных по поводу ПГПТ в КВМТ им. Н.И. Пирогова СПбГУ в 2017–2018 гг. В исследование было включено 810 случаев. Оценивались демографические показатели, данные предоперационного обследования и непосредственные результаты хирургического лечения, была построена логистическая регрессионная модель для оценки риска множественного поражения околощитовидных желез. Сравнивалась частота персистенции ПГПТ и случаев множественного поражения между группами с конкордантными и дискордантными результатами визуализации.РЕЗУЛЬТАТЫ. Возраст, пол, индекс массы тела, негативные результаты предоперационного ультразвукового исследования, сцинтиграфии и рентгеновской компьютерной томографии в качестве независимых переменных оказались не ассоциированы с риском множественного поражения. Большее число выполненных предоперационных исследований оказалось связано с риском персистенции ПГПТ. 37% случаев множественного поражения не было выявлено на дооперационном этапе. Выявлено 7 случаев с дополнительными аденомами, обнаруженными только благодаря выполнению двусторонней ревизии шеи.ЗАКЛЮЧЕНИЕ. На данный момент любая комбинация методов предоперационной визуализации не позволяет надежно выявлять множественное поражение околощитовидных желез. Выполнение дополнительных визуализирующих исследований не приводит к повышению эффективности хирургического лечения. Выполнение двусторонней ревизии шеи может привести к снижению частоту персистенции ПГПТ благодаря лучшему выявлению случаев множественного поражения.

## Abstract (en)

BACKGROUND: Precise localization of abnormal parathyroid glands is important for a successful surgery for primary hyperparathyroidism (PHPT). While a large number of patients can be successfully treated with the focused parathyroidectomy, there is a considerable rate of the persistent PHPT mostly because of undetected multiglandular disease (MGD).AIM: The aim of the study was to evaluate the meaning of preoperative visualization data for planning the surgery for patients with PHPT.MATERIALS AND METHODS: The study was conducted at SPBU Hospital in 2017-2018. 810 patients who underwent a primary surgery for PHPT were included in the study. Preoperative imaging results were investigated and multivariative logistic regressions were calculated to assess the predictive values of preoperative data. The rate of cases with persistent disease and cases with MGD were compared between patients with different results of preoperative data.RESULTS: Age, sex, body mass index, negative results of preoperative US, MIBI and 4D CT were not independently associated with the higher risk of multiglandular disease. The larger number of performed preoperative visualization studies were associated with the higher risk of persistence. 37% cases of MGD were not identified preoperatively. There were 7 cases with previously unsuspected second adenomas found only due to bilateral neck exploration.CONCLUSION: Any combination of preoperative visualization modalities was not able to rule out the MGD reliably. Efficacy of surgical treatment was not associated with the higher number of preoperative studies. Bilateral neck exploration may decrease the rate of the persistent hyperparathyroidism improving the identification of multiglandular disease.

## ОБОСНОВАНИЕ

Число операций по поводу первичного гиперпаратиреоза (ПГПТ) неуклонно растет, поскольку хирургическое лечение является единственным методом полного излечения данного заболевания, безопасно, а также имеет преимущества перед консервативным лечением с точки зрения экономической эффективности [1][2].

Большинство пациентов с ПГПТ могут быть излечены с помощью селективной паратиреоидэктомии, основанной на результатах предоперационной визуализации. Сцинтиграфия с Tc99-сестамиби (технетрилом) в сочетании с ультразвуковым исследованием считается «золотым стандартом» визуализации околощитовидных желез (ОЩЖ) [3]. Селективные и минимально инвазивные операции за последнее десятилетие стали основным методом лечения пациентов с ПГПТ, сменив традиционно выполнявшуюся ранее ревизию шеи, показывая отличную эффективность одновременно с меньшей частотой осложнений и более коротким временем операции [4].

Тем не менее риск послеоперационной персистенции гиперпаратиреоза при выполнении селективной паратиреоидэктомии составляет до 3% [5] — во многом из-за наличия пациентов с множественным поражением околощитовидных желез, не выявляемым при предоперационном обследовании.

Большая часть случаев ПГПТ обусловлена наличием единственной аденомы ОЩЖ, однако множественное поражение может встречаться достаточно часто (от 3 до 33%, по разным данным) [6]. Множественное поражение ОЩЖ может иметь как спорадический, так и наследственный характер [7].

В качестве дополнительного метода при получении дискордантных результатов по данным сцинтиграфии и УЗИ используется рентгеновская компьютерная томография (КТ) с внутривенным болюсным контрастированием [2]. При этом КТ также показывает высокую диагностическую точность (около 93%) в качестве метода первой линии [8], но обладает относительно невысокой предсказательной способностью в отношении множественного поражения [3].

Однофотонная эмиссионная томография имеет меньшую частоту ложноотрицательных результатов по сравнению с планарной сцинтиграфией (чувствительность метода — 74%), но не отличается по прогностической ценности положительного результата [9].

Ожидается, что развитие технологий предоперационной визуализации улучшит поиск аденом ОЩЖ и позволит более надежно планировать выполнение селективной паратиреоидэктомии. Однако на данный момент до 30% случаев множественного поражения остаются нераспознанными на предоперационном этапе [3][10].

В качестве альтернативы селективной паратиреоидэктомии рассматривается рутинное выполнение двусторонней ревизии шеи (ДРШ) с визуализацией всех четырех ОЩЖ [11, 12], но подобный вид операции имеет более высокие требования к хирургической технике, грозит большим числом возможных осложнений и по-прежнему вызывает сомнения с точки зрения экономической эффективности [13][14].

## ЦЕЛЬ ИССЛЕДОВАНИЯ

Целью настоящего исследования являлось изучение возможностей предоперационной визуализации ОЩЖ при планировании объема хирургического вмешательства при лечении больных с ПГПТ.

## МАТЕРИАЛЫ И МЕТОДЫ

## Место и время проведения исследования

Место проведения. КВМТ им. Н.И. Пирогова СПбГУ.

Время исследования. Январь 2017 — декабрь 2018 г.

## Изучаемая популяция

Пациенты, получавшие хирургическое лечение по поводу ПГПТ в указанный период времени.

Критерии включения: лабораторно подтвержденный диагноз ПГПТ.

Критерии исключения: наличие в анамнезе операций по поводу ПГПТ.

## Способ формирования выборки из изучаемой популяции

Способ формирования выборки — сплошной.

## Дизайн исследования:

## Методы

Уровень ионизированного кальция в сыворотке крови исследовался до операции и на следующий день после операции. Уровень паратгормона (ПТГ) оценивали до операции, в течение 1 ч после операции и на следующий день после операции. Концентрация ионизированного кальция измерялась с помощью технологии ISE с использованием анализатора EasyLyte Calcium-MEDICA (референсный диапазон 1,13–1,31 ммоль/л). Уровень ПТГ определялся методом иммунохемилюминесцентного анализа с использованием анализатора Beckman Coulter DXI800 (референcный диапазон составлял 1,3–9,3 пмоль/л). Все пациенты, которым был установлен диагноз нормокальциемического ПГПТ, до этого получали препараты витамина D и имели сохраненную функцию почек.

Данные по визуализирующим исследованиям записывались как категориальные переменные: было или не было выполнено исследование; визуализирован хотя бы один объект как увеличенная ОЩЖ или нет; для каждой из четырех ОЩЖ: визуализирована или нет, неопределенные результаты расценивались как отрицательные.

У лечащего врача была возможность дополнительно назначить КТ шеи перед операцией, но не радиоизотопное исследование, что обусловлено техническими возможностями клиники.

ДРШ считалась операция с попыткой визуализации всех ОЩЖ. Операция с удалением любых двух ОЩЖ, выполненная на основании данных предоперационной визуализации, без указания в протоколе операции на попытку поиска остальных ОЩЖ не считалась ДРШ. Показанием к выполнению ДРШ являлось подозрение на множественное поражение или отсутствие локализованной аденомы по результатам предоперационной визуализации. Также ДРШ могла выполняться в случае наличия одной предоперационно локализованной аденомы ОЩЖ, в этом случае такая тактика не была регламентирована и оставлялась на усмотрение хирурга. Алгоритм ДРШ не был унифицирован. Интраоперационное измерение уровня ПТГ не проводилось.

Персистенция гиперпаратиреоза определялась как уровень ионизированного кальция выше верхней границы нормы (1,31 ммоль/л) при повышенном (или не подавленном при нормогормональном ПГПТ) уровне ПТГ на следующий день после операции. Для нормокальциемического ПГПТ персистенция определялась как повышенный уровень ПТГ на следующий день после операции при уровне ионизированного кальция в верхней половине референсного интервала. Период наблюдения был ограничен временем пребывания пациента в стационаре. Случаи множественного поражения был определены следующим образом: 1) гистологически подтвержденное удаление более чем одной аденомы за период текущей госпитализации; 2) удаление более одной увеличенной железы, ни одна из которых не была описана гистологически как аденома (описана гиперплазия ОЩЖ), но приведшее к разрешению гиперпаратиреоза; 3) персистенция ПГПТ после удаления одной гистологически подтвержденной аденомы.

## Статистический анализ

Критерий хи-квадрат Пирсона использовался для сравнения категориальных переменных. Была построенная логистическая регрессионная модель для оценки риска персистенции и риска множественного поражения. В анализ были включены следующие независимые переменные: возраст, пол, индекс массы тела, отрицательный результат сцинтиграфии (да или нет), отрицательный результат УЗИ (да или нет), отрицательный результат КТ (да или нет), количество выполненных предоперационных визуализирующих исследований (1, 2 или 3), наличие операций на шее в анамнезе (да или нет). Были рассчитаны отношения шансов (ОШ) и 95% доверительные интервалы (ДИ). Значение p<0,05 считалось статистически значимым.

Статистическая обработка данных производилась с помощью языка программирования R в RStudio Version 1.3.1056.

## Этическая экспертиза

Заключение комитета по биомедицинской этике Клиники высоких медицинских технологий им. Н.И. Пирогова СПбГУ вх. №100 от 11.04.2022: учитывая ретроспективный характер исследования и отсутствие персональных идентификационных данных, неинтервенционный дизайн исследования, письменного согласия пациентов и специального одобрения этическим комитетом не требуется.

## РЕЗУЛЬТАТЫ

Медиана возраста пациентов составила 58 лет (9–87). Из них 6,1% —мужчины, 93,9% — женщины. ДРШ в качестве первичной операции была выполнена в 200 случаях (24,7%). В 6 случаях одновременно с операцией по поводу гиперпаратиреоза производилась тиреоидэктомия, в 22 случаях — гемитиреоидэктомия.

Всего выявлено 43 случая послеоперационной персистенции гиперпаратиреоза (5,3%). Были выявлены следующие причины персистенции ПГПТ: 1) удалены не все аденомы ОЩЖ (n=24); 2) вместо аденомы удалена нормальная ОЩЖ или ОЩЖ спризнаками гиперплазии (n=13); 3) не удалена ни одна ОЩЖ (n=7).

В выборку вошел 21 случай ПГПТ со стойкой нормокальциемией. Во всех случаях локализация увеличенной ОЩЖ была установлена по данным хотя бы одного метода визуализации. В 19 из них после операции зафиксировано падение уровня паратгормона более чем в два раза, при гистологическом исследовании в 17 случаях подтверждено удаление аденомы ОЩЖ, в 2 — описаны ОЩЖ с признаками гиперплазии. Еще в 2 случаях в послеоперационном периоде была отмечена персистенция гиперпаратиреоза, вызванная наличием второй аденомы, в обоих случаях после удаления второй аденомы за период текущей госпитализации наступило разрешение гиперпаратиреоза.

Общее количество случаев множественного поражения составило 54 (6,67%). 5 из них были идентифицированы как пациенты с множественной эндокринной неоплазией 1-го типа к моменту сбора данных.

Была построена логистическая регрессионная модель с несколькими переменными для оценки риска множественного поражения ОЩЖ. Среди оцениваемых переменных значимых предикторов обнаружено не было. Оценивались отрицательные результаты предоперационной визуализации, индекс массы тела, пол и возраст (табл. 1).

**Table:** Таблица 1. Показатели отношения шансов и 95% доверительных интервалов независимых переменных регрессионной модели для оценки риска множественного поражения ОЩЖTable 1. Odds ratios and 95% confidence intervals of independent variables of the regression model for assessing the risk of multiple PTG lesions

Показатели	ОШ	2,5%	97,5%
Негативные результаты УЗИ	1,122	0,440	2,861
Негативные результаты КТ	4,282	0,848	21,627
Негативные результаты сцинтиграфии	1,243	0,407	3,797
ИМТ	1,000	0,983	1,017
Пол	1,26	0,.373	4,258
Возраст	0,987	0,.964	1,011

Во всех случаях до операции оперирующим хирургом было выполнено УЗИ шеи. В 109 случаях было выполнено только УЗИ. В 356 случаях на догоспитальном этапе выполнена планарная сцинтиграфия с технетрилом. В 181 случае КТ с внутривенным введением контраста была выполнена вместо сцинтиграфии на догоспитальном этапе или во время пребывания в стационаре. В 164 случаях КТ была дополнительно выполнена в качестве третьего визуализирующего исследования.

Разные методики визуализации независимо друг от друга показали чувствительность от слабой до умеренной в отношении выявления множественного поражения ОЩЖ, при этом КТ и сцинтиграфия имели значимо лучшую чувствительность, чем УЗИ (табл. 2).

**Table:** Таблица 2. Диагностические показатели различных методов визуализацииTable 2. Diagnostic performance of various imaging modalities Примечание. ПЦПР — прогностическая ценность положительного результата; ПЦОР — прогностическая ценность отрицательного результата.

	УЗИ	Сцинтиграфия	КТ
Значение, %	95% ДИ	Значение, %	95% ДИ	Значение, %	95% ДИ
Чувствительность	22,92	12,03–37,31	33,33	17,29–52,81	62,16	44,76–77,54
Специфичность	97,99	96,65–98,90	92,69	89,78–94,98	90,13	86,21–93,24
ПЦПР	44,00	27,39–62,07	24,39	14,93–37,23	43,40	33,44–53,92
ПЦОР	94,85	94,04–95,56	95,16	93,84–96,20	95,14	92,82–96,74

Помимо УЗИ, в 548 случаях была выполнена сцинтиграфия либо КТ шеи с внутривенным контрастированием, а в 156 случаях — все три исследования. Пациенты, которым было выполнено большее число исследований, имели значимо большую частоту множественного поражения, также в этих группах значимо чаще встречалась персистенция гиперпаратиреоза после операции (табл. 3).

**Table:** Таблица 3. Число случаев множественного поражения ОЩЖ и персистенции ПГПТ в группах с различным числом выполненных предоперационных исследованийTable 3. The number of cases of multiple PTG lesions and persistent PHPT in groups with different numbers of preoperative studies performed Примечание: *различия статистически значимы при p<0,05.

Выполнено исследований	3	2	Только УЗИ
Количество пациентов	156	548	109
Множественное поражение	21	32	1
Частота множественного поражения, %	13,5*	5,8*	0,9*
Случаев персистенции	15	27	1
Частота персистенции, %	9,6*	4,9*	0,9*

В 673 случаях предоперационно имелось указание на наличие только одной аденомы по данным как минимум одного метода визуализации. В 137 случаях аденома либо не была визуализирована, либо имелись указания на наличие более чем одной аденомы, либо результаты нескольких исследований были дискордантны. Частота множественного поражения ОЩЖ и общая частота персистенции ПГПТ были значимо выше во 2-й группе, в то время как частота персистенции ПГПТ из-за нераспознанного множественного поражения значимо не различалась между группами (табл. 4). В 131 случае ДРШ была выполнена пациентам, у которых на предоперационном этапе по результатам как минимум одного исследования была обнаружена одна аденома, при этом в 3 случаях интраоперационно была обнаружена вторая аденома ОЩЖ. В данной группе пациентов персистенция ПГПТ не встречалась. Таким образом, ни одного случая множественного поражения ОЩЖ в группе с ДРШ не было пропущено.

**Table:** Таблица 4. Число случаев множественного поражения ОЩЖ и персистенции ПГПТ в группе с одной визуализированной аденомой и группе с негативными либо дискордантными результатами или несколькими визуализированными аденомамиTable 4. Number of cases of multiple PTG lesions and persistent PHPT in the group with one visualized adenoma and the group with negative or discordant results or multiple visualized adenomas Примечание: p<0,05

	Визуализирована одна аденома	Аденома не визуализирована, визуализировано более одной потенциальной аденомы либо результаты разных методов дискордантны	Хи-квадрат	p
Число пациентов	673	137		
Число пациентов с множественным поражением	22	32		
Частота множественного поражения, %	3,3	23,4	73,826	<0,001*
Число пациентов с персистенцией гиперпаратиреоза	28	15		
Частота персистенции гиперпаратиреоза, %	4,2	10,9	10,435	0,002*
Число пациентов с персистенцией гиперпаратиреоза из-за пропущенного множественного поражения	19	5		
Частота персистенции гиперпаратиреоза из-за пропущенного множественного поражения, %	2,8	3,6	0,270	0,604
ДРШ выполнено	131	69		

Пациенты, которым было выполнено два или три визуализирующих исследования, были разделены на 2 группы. В 1-ю группу вошли пациенты с полностью конкордантными результатами визуализации, то есть имеющие не менее двух предоперационных визуализирующих исследований, каждое из которых указывало на одну аденому ОЩЖ в одном и том же месте (n=461). Все остальные случаи с негативной визуализацией, случаи с частично конкордантными результатами и случаи с дискордантными результатами были отнесены ко 2-й группе (n=240).

И множественное поражение ОЩЖ, и персистенция ПГПТ значимо чаще встречались в группе с дискордантными результатами, в то время как частота персистенций, вызванных пропущенным множественным поражением, значимо не различалась (табл. 5). В 87 случаях пациентам с конкордантными результатами предоперационной визуализации была выполнена ДРШ, в 2 случаях интраоперационно была обнаружена вторая аденома.

**Table:** Таблица 5. Число случаев множественного поражения ОЩЖ и персистенции ПГПТ в группе с полностью конкордантными и группе с неидеальными результатами визуализацииTable 5. Number of cases of multiple PTG lesions and persistence of PHPT in the fully concordant group and the suboptimal imaging group Примечание: p<0,05

	Полностью конкордантные результаты визуализации	Негативные, дискордантные или частично конкордатные результаты визуализации	Хи-квадрат	p
Число пациентов	461	240		
Число пациентов с множественным поражением	15	39		
Частота множественного поражения, %	3,3	16,3	37,494	<0,001*
Число пациентов с персистенцией гиперпаратиреоза	18	23		
Частота персистенции гиперпаратиреоза, %	3,9	9,6	9,243	0,003*
Число пациентов с персистенцией гиперпаратиреоза из-за пропущенного множественного поражения	13	11		
Частота персистенции гиперпаратиреоза из-за пропущенного множественного поражения, %	2,8	4,6	1,484	0,224
ДРШ выполнено	87	88		

Общее количество пациентов с хотя бы одной аденомой, не обнаруженной до операции при проведении не менее двух исследований, составило 46. В 30 случаях из 46 была проведена ДРШ, в результате чего в 27 случаях (90%) была обнаружена аденома, не локализованная ранее.

В 20 случаях на предоперационном этапе была визуализирована только одна аденома из двух имеющихся, что составило 37% общего числа случаев множественного поражения. Всего было 7 случаев с ранее не подозревавшимися вторыми аденомами, обнаруженными только благодаря ДРШ.

После исключения случаев с одновременным выполнением вмешательства на щитовидной железе в обеих группах (с выполнением ДРШ (n=187) и без нее (n=595)) не было случаев послеоперационных кровотечений. В группе без ревизии отмечено 19 случаев одностороннего пареза гортани (595 нервов под риском), 7 — в группе с выполнением ДРШ (374 нерва под риском). В группе ДРШ не отмечено случаев двусторонних парезов гортани и парезов с той стороны, где не производилось удаление аденомы ОЩЖ. В группе с ДРШ выявлено 15 случаев гипокальциемии в сочетании с низким и низконормальным уровнем ПТГ, в группе без выполнения ДРШ — 42 случая (хи-квадрат 0,195, p=0,659).

## ОБСУЖДЕНИЕ

Как показывает проведенное нами исследование, на данный момент любой из рутинных методов предоперационной визуализации либо их комбинация не позволяют надежно исключить множественное поражение ОЩЖ и отобрать тех пациентов, которым для излечения будет достаточно выполнения селективной паратиреоидэктомии. Даже в группе пациентов с полностью конкордантными результатами визуализации число случаев множественного поражения составляет 3,3%, при этом в данной группе встречается почти треть общего числа случаев множественного поражения ОЩЖ. Это означает, что отсутствие ревизии всех ОЩЖ приведет к рецидиву ПГПТ в 1 случае из 30 операций в группе пациентов, где результаты визуализирующих методов создают впечатление об уверенности в локализации аденомы ОЩЖ.

Более строгие требования к интерпретации разных методов визуализации улучшают выявление аденом, но не позволяют надежно исключить множественное поражение. Эти данные могут указывать на возможно более широкое применение ДРШ в группе пациентов с указанием на единственную аденому по данным предоперационного обследования. Применение ДРШ, согласно актуальным клиническим рекомендациям, ограничено случаями с отсутствием локализованной аденомы по данным визуализирующих исследований, а также при подозрении на множественное поражение ОЩЖ [15].

Несмотря на это, рядом исследователей отмечается тренд возрастающей частоты выполнения ДРШ. Одним из возможных объяснений называется возрастание доли пациентов с мягкими формами гиперпаратиреоза, ассоциированными с меньшими размерами аденом и большей частотой негативных результатов визуализации [16].

В метаанализе 2017 г., охватившем 12 743 пациента из 19 исследований, не выявлено статистически значимой разницы в частоте персистенции и рецидивов гиперпаратиреоза, а также в частоте выполнения повторных операций. Не было выявлено различий в частоте осложнений за исключением транзиторной гипокальциемии (1,6% в группе селективной паратиреоидэктомии и 13,2% в группе с ДРШ) [17].

В отличие от других работ [18] нам не удалось выявить ассоциацию негативных результатов сцинтиграфии с повышенным риском множественного поражения. По-видимому, отсутствие накопления радиофармпрепарата по данным сцинтиграфии чаще бывает вызвано небольшим размером аденомы, нежели равномерным распределением между несколькими гиперфункционирующими ОЩЖ. Это особенно важно в контексте того, что типичная клиническая картина заболевания в настоящее время встречается реже, и значительную долю занимают пациенты с мягким течением гиперпаратиреоза, нормокальциемическим ПГПТ и ПГПТ со стойко нормальным уровнем ПТГ. В странах Европы и Северной Америки частота манифестных форм ПГПТ не превышает 20%. В России за последние годы доля мягкой формы ПГПТ также растет, хотя, по данным 2012 г., ее частота составляет не более 28%, что значимо меньше по сравнению с данными зарубежных исследований [19]. Соответственно, чаще причиной заболевания являются аденомы ОЩЖ небольшого размера, что затрудняет дооперационную топическую диагностику [20].

Дополнительная предоперационная визуализация (КТ в качестве метода 3-й линии после УЗИ и сцинтиграфии), по-видимому, имеет ограниченную эффективность в отношении снижения риска персистенции. Отчасти это может быть объяснено эффектом «сложного» случая. Пациенты с небольшими аденомами, которые не были видны при стандартной визуализации, получали более широкое предоперационное обследование наряду с более тщательным выполнением интраоперационной ревизии, в то время как «простым» случаям уделялось меньше внимания в отношении риска множественного поражения. Так, в группе с дискордантными результатами ДРШ была выполнена у 36,7% пациентов, в то время как в группе с конкордантными результатами — только в 18,9%. Вероятно, поэтому дискордантные результаты предоперационной визуализации не были ассоциированы с более высоким риском персистенции, вызванной множественным поражением, что можно было бы предполагать.

Ограничением исследования является его ретроспективный характер. Так, большое количество случаев, когда ДРШ не была выполнена в группе с дискордантными результатами предоперационной визуализации, может свидетельствовать о том, что ретроспективная оценка результатов предоперационной визуализации не всегда совпадает с их интерпретацией клиницистом в процессе лечения.

Также невозможно однозначно установить видение хирургом клинической ситуации и факторы, которые могут оказать влияние на принятие решения о выполнении или невыполнении ДРШ в условиях, когда показания строго не регламентированы. Это во многом связано с тем, что все методы визуализации являются операторзависимыми методами. В рамках текущего исследования сцинтиграфия выполнялась в разных учреждениях, разнородность в качестве проведения исследования иинтерпретации результатов накладывает ограничение на сделанные выводы о недостатках этого метода.

Вероятность ошибок в интерпретации результатов предоперационной визуализации может быть снижена при участии в обсуждении результатов диагностики мультидисциплинарной команды с вовлечением специалистов-радиологов. Однако даже в этом случае есть риск, что хирург может отказаться от выполнения ДРШ, если оно не было заранее запланировано, после удаления одной аденомы, только частично удовлетворяющей результатам предоперационной визуализации.

В рамках данного исследования невозможно оценить качество выполненных ревизий: поскольку рутинная биопсия визуально неизмененных ОЩЖ не производилась, истинная частота визуализации полного комплекта ОЩЖ остается неизвестной.

Низкая в целом частота послеоперационной персистенции гиперпаратиреоза и случаев множественного поражения, вероятно, требует большей выборки пациентов для выявления ассоциированных с ними факторов риска.

## ЗАКЛЮЧЕНИЕ

В настоящее время любая комбинация методов предоперационной визуализации, доступных для массового применения, не позволяет надежно выявить случаи множественного поражения ОЩЖ. Увеличение числа выполняемых пациенту исследований не приводит к повышению эффективности хирургического лечения (уменьшению частоты персистенции гиперпаратиреоза). Рутинное выполнение двусторонней ревизии шеи может привести к снижению частоты персистенции ПГПТ засчет лучшего выявления случаев множественного поражения и может быть рекомендовано для клиник, выполняющих большое количество операций по поводу ПГПТ.

## ДОПОЛНИТЕЛЬНАЯ ИНФОРМАЦИЯ

Источники финансирования. Работа выполнена по инициативе авторов без привлечения финансирования.

Конфликт интересов. Авторы декларируют отсутствие явных и потенциальных конфликтов интересов, связанных с содержанием настоящей статьи.

Участие авторов. Бузанаков Д.М., Семенов А.А., Черников Р.А., Слепцов И.В., Тимофеева Н.И., Карелина Ю.В., Новокшонов К.Ю. — идея и дизайн исследования; Семенов А.А., Черников Р.А., Слепцов И.В., Тимофеева Н.И., Карелина Ю.В., Новокшонов К.Ю. — предоставление материалов исследования; Бузанаков Д.М., Джуматов Т.А., Яневская Л.Г. — сбор данных, формирование выборки пациентов; Бузанаков Д.М., Джуматов Т.А. — формирование и ведение базы данных; Бузанаков Д.М., Джуматов Т.А., Яневская Л.Г. — анализ и интерпретация данных, написание текста рукописи; Семенов А.А., Черников Р.А., Слепцов И.В., Тимофеева Н.И., Яневская Л.Г., Карелина Ю.В., Новокшонов К.Ю. — финальный анализ и редактирование текста рукописи. Все авторы одобрили финальную версию статьи перед публикацией, выразили согласие нести ответственность за все аспекты работы, подразумевающую надлежащее изучение и решение вопросов, связанных с точностью или добросовестностью любой части работы.
